# Feasibility of crude F4 fimbriae extract as a vaccine candidate for preventing *Escherichia coli*-induced diarrhea in piglets

**DOI:** 10.14202/vetworld.2023.2063-2070

**Published:** 2023-10-07

**Authors:** Luong Thi Yen Nguyet, Puey Ounjai, Kampon Kaeoket, Natharin Ngamwongsatit

**Affiliations:** 1 Department of Clinical Sciences and Public Health, Faculty of Veterinary Science, Mahidol University, Nakhon Pathom 73170, Thailand; 2 Department of Biology, Faculty of Science, Mahidol University, Bangkok 10400, Thailand; 3 Laboratory of Bacteria, Veterinary Diagnostic Center, Faculty of Veterinary Science, Mahidol University, Nakhon Pathom 73170, Thailand

**Keywords:** diarrhea, *Escherichia coli*, F4 fimbriae, piglets, vaccine

## Abstract

**Background and Aim:**

Enterotoxigenic *Escherichia coli* (ETEC) poses a substantial risk of neonatal diarrhea and post-weaning diarrhea among piglets, with F4^+^ ETEC strains emerging as a particularly challenging issue within the pig farming industry. This study aimed to introduce a straightforward approach for generating a crude extract of F4 fimbriae that shows promise as an antigenic determinant for potential vaccination strategies.

**Materials and Methods:**

A crude F4 fimbriae extract was obtained from F4^+^ ETEC using a combination of heat shock and homogenization techniques. Subsequently, three 4-week-old piglets were immunized with a primary dose of 150 μg and a booster dose 2 weeks later. Blood samples were collected to evaluate the level of serum F4-specific antibodies using an enzyme-linked immunosorbent assay.

**Results:**

Analysis using sodium dodecyl sulfate-polyacrylamide gel electrophoresis and liquid chromatography tandem-mass spectrometry techniques unveiled crucial insights into the composition of the crude F4 fimbriae extract. Notably, a distinct prominent band (~24 kDa) was identified, corresponding to the size of FaeG, the major subunit of F4 fimbriae. Regarding antibody response, there was a remarkable disparity between the levels of serum immunoglobulin (Ig)G and IgA antibodies targeting F4 compared with other *E*. *coli* strains (F18^+^ ETEC, F41^+^ ETEC, and F4^−^F18^−^F41^−^ EC), as well as with the unvaccinated control group (p < 0.01). Specifically, the levels of IgG antibodies against other *E*. *coli* strains were also significantly higher than those observed in the unvaccinated control group (p < 0.05).

**Conclusion:**

Our findings suggest that the crude F4 fimbriae extracts obtained using our simple extraction method induce specific immune responses against F4^+^ *E. coli* and stimulate cross-immunity against other *E*. *coli* strains. Therefore, our method shows potential for use in future vaccine development against diarrhea in pigs caused by *E*. *coli*.

## Introduction

Enterotoxigenic *Escherichia coli* (ETEC) infection is a serious concern in the swine farming industry, causing neonatal diarrhea and post-weaning diarrhea (PWD), with high morbidity and mortality rates in piglets. This results in significant losses to the global livestock industry [1, 2]. The occurrence of diarrhea depends on interactions between the causative bacteria and environmental conditions, the health status of the animal, and the occurrence of multidrug-resistant bacteria in pig herds [3, 4]. Enterotoxigenic *E. coli* initiates infection by attaching to specific receptors in the epithelium of the small intestine of pigs using fimbrial adhesins. This attachment is followed by the production of enterotoxins (Sta, Stb, and LT) that cause severe electrolyte imbalance, leading to diarrhea [5]. The two main virulence factors in ETEC pathogenesis are enterotoxins and fimbrial adhesins [6]. Fimbriae are long proteinaceous appendages with a diameter of approximately 2–4 nm that project from the surface of ETEC [7]. Various fimbrial proteins have been proposed as associated with ETEC pathogenesis in swine, including F4 (K88), F5 (K99), F6 (987P), F18, and F41 [2]. Enterotoxigenic *E. coli* strains displaying F5, F6, and F41 fimbriae usually cause neonatal diarrhea in newborn piglets and can be effectively controlled by vaccinating pregnant sows [8]. Fimbrial proteins show high immunological activity and can induce mucosal and systemic immune responses in piglets [9]. However, the levels of maternal antibodies that protect piglets from ETEC-associated neonatal diarrhea usually drop quickly after weaning [6]. Thus, F4^+^ and F18^+^ ETEC infections commonly occur post-weaning (2–6 weeks after weaning) [8]. Moreover, F4^+^ ETEC also causes neonatal diarrhea, resulting in a significant financial burden on the pig production system [8]. Therefore, controlling infections caused by F4^+^ ETEC is crucial for managing ETEC transmission [10, 11].

The major subunit of F4 adhesion machinery is the FaeG protein (~24 kDa) [12]. It was reported that not only purified F4 fimbriae but also recombinant FaeG protein could be successfully exploited to stimulate immune protection in piglets against F4^+^ ETEC-related diarrhea [7]. Unfortunately, the high cost of fimbriae purification has limited the potential profit and its application. In addition, the antibodies formed in response to vaccination were often reported as only specific for the particular fimbrial antigen present in the vaccine. Furthermore, cross-immunity among heterogeneous fimbrial antigens has yet to be elucidated [13, 14].

Our simplified protocol for partially purifying F4 fimbriae has the potential to significantly reduce vaccine production costs, making it more cost-effective. Moreover, piglets vaccinated with crude F4 extracts displayed a broad-spectrum immune response against various types of ETEC fimbriae, demonstrating its potential as an alternative approach for preventing ETEC infection.

This study aimed to assess the feasibility of employing partially purified F4 fimbriae obtained through a simple protocol as a candidate for vaccine development against neonatal diarrhea and PWD caused by *E*. *coli*.

## Materials and Methods

### Ethical approval

The animal experiments were approved by the Faculty of Veterinary Science-Animal Care and Use Committee (FVSACUC-Protocol No. MUVS-2019-06-31 and MUVS-2021-10-40).

### Study period and location

This study was conducted from December 2021 to June 2022. The samples were collected fromfarms at Nakhon Pathom and analyzed at Veterinary Diagnosis Center, Faculty of Veterinary Science, Mahidol University, Nakhon Pathom, Thailand.

### Bacterial strains

We utilized four pathogenic *E. coli* isolates: EC5W7LF (F4^+^ ETEC), MI1068B/61LF (F18^+^ ETEC), MI907/62 (F41^+^ ETEC), and MI664-2LF (non-fimbrial *E. coli* strain: F4^−^F18^−^F41^−^ EC). The isolates were obtained from rectal swabs of diarrheic piglets on farms in Thailand during an edema disease outbreak from 2018 to 2019 [15]. Bacterial stocks were stored at −80°C at the Veterinary Diagnosis Center (Faculty of Veterinary Science, Mahidol University, Nakhon Pathom, Thailand) until use. All isolates containing genes associated with fimbriae were validated by multiplex polymerase chain reaction assay, as previously described by Nguyet *et al*. [15].

### Extraction and identification of crude F4 fimbriae from F4^+^ ETEC

Crude F4 fimbriae were extracted using a modified protocol combining heat shock and homogenization, as per Vazquez *et al*. [16] and Van den Broeck *et al*. [17]. First, a colony of F4^+^ ETEC was cultured on blood agar, inoculated into 20 mL of brain heart infusion (BHI) broth, and incubated overnight at 37°C with agitation at 1.36× *g*. Then, the culture was scaled up by inoculating 1 mL of the overnight culture into flasks containing 300 mL of BHI broth, which were incubated under the same conditions. The cells were harvested by centrifugation at 10,000× *g* for 10 min and washed twice with phosphate-buffered saline (PBS; pH 7.2). Next, the cells from each 300 mL of culture were suspended in 6 mL of PBS (pH 7.2). To release the fimbriae, the suspension was heat-treated at 60°C for 25 min in a water bath, followed by homogenization for 10 min using a homogenizer. After centrifugation at 12,000× *g* for 30 min at 4°C, the supernatant containing the crude F4 fimbriae extract was collected. Then, the cell pellets were resuspended in PBS (pH 7.2) and subjected to a final round of fimbriae extraction as described above. The protein concentration was determined using a Bradford protein assay [18]. The molecular weights of the components of the crude fimbriae extracts were verified using sodium dodecyl sulfate-polyacrylamide gel electrophoresis (SDS-PAGE), and a protein of ~24 kDa was identified using a Triple-TOF 6600 + liquid chromatography tandem-mass spectrometry (LC-MS/MS) system (Triple-TOF^®^ 6600 + LC-MS/MS System, Sciex, Framingham, MA, USA). The crude F4 fimbriae extract and residual F4^+^ ETEC were inactivated with 0.1% formaldehyde (Formaldehyde solution about 37%, Merck, Darmstadt, Germany) and stored at −20°C until use for vaccine production.

### Stimulating piglets’ immune system with crude F4 fimbriae extracts

The animal experiment in this study utilized six piglets divided into a vaccinated group and an unvaccinated (control) group (n = 3 per group). The vaccine was prepared by mixing our crude F4 fimbriae antigen (150 μg/mL) with an equal volume of adjuvant (Montanide™ ISA 206 VG, Seppic, Paris, France) following the manufacturer’s instructions to form a stable emulsion. The primary immunization was administered to 4-week-old piglets (n = 3) via intramuscular injection in the neck with 2 mL of the vaccine (containing 150 μg of antigen). Two weeks later, a booster immunization containing the same vaccine dose through the same route as before was administered. Blood samples (~5 mL) were collected from the jugular vein before vaccination and 2 weeks after the second dose. All blood samples were centrifuged at 3,000× *g* for 10 min at 4°C, and the serum was collected and stored at −20°C for later use. Blood samples were collected from the unvaccinated group (n = 3) and treated in the same manner as those collected from the vaccinated group to obtain an unvaccinated serum control.

### Preparation of inactivated whole-cell *E. coli* for enzyme-linked immunosorbent assay (ELISA)

Overnight cultures of *E*. *coli* strains, including EC5W7LF (F4^+^ ETEC), MI1068B/61LF (F18^+^ ETEC), MI907/62 (F41^+^ ETEC), and MI664-2LF (F4^−^F18^−^F41^−^ EC), were prepared and inactivated with 0.1% formaldehyde (Formaldehyde solution approximately 37%, Merck) at 37°C for 1 h with shaking. Then, the inactivated cells were incubated at 4°C for 18 h as previously described by Arshadi *et al*. [19], collected by centrifugation at 10,000× *g* or 10 min at 4°C, and washed twice with sterile PBS (pH 7.2). Finally, the cell pellet was resuspended in PBS (pH 7.2) to a concentration of 10^10^ colony-forming unit (CFU)/mL and stored at −4°C until use.

### Determination of the level of specific antibodies in pig serum by ELISA

To evaluate the immune response in the piglets immunized with our crude extract, we used a modified ELISA protocol to determine the presence of total immunoglobulin (Ig)G and IgA antibodies against F4 fimbriae or other inactivated *E. coli* antigens in the collected serum samples [6]. Briefly, 96-well microtiter plates were coated with coating buffer (0.1 M carbonate buffer, pH 9.4) containing a compatible concentration of each antigen, including F4 fimbriae antigen (0.5 μg/well) or inactivated F4^+^ ETEC, F18^+^ ETEC, F41^+^ ETEC, or F4^−^F18^−^F41^−^ EC cells (10^7^ CFU/well) and incubated overnight at 4°C. Then, the coated plates were washed 3 times with PBS containing 0.05% Tween 20 (PBST), blocked with PBST containing 3% bovine serum albumin, and incubated at 37°C in a humidity chamber for 2 h. Each serum sample underwent twofold serial dilution (starting at 1:250) in PBST, and duplicate samples were added to the coated wells. Then, the plates were incubated at 37°C in a humidity chamber for 1 h. After washing 3 times with PBST, 1:20,000 dilutions of goat anti-pig IgG horseradish peroxidase (HRP) antibody (Abcam, Cambridge, England) or goat anti-pig IgA HRP antibody (Abcam) in PBST were added to each well (50 μL/well), and the plates were incubated at 37°C in a humidity chamber for 1 h. The plates were washed again, and 3,3',5,5'-Tetramethylbenzidine Liquid Substrate System for ELISA (Sigma-Aldrich, Darmstadt, Germany) was added (50 μL/well). Then, the plates were incubated in the dark at room temperature (~25ºC) for 10–15 min, and the reaction was stopped by adding 1 M H_2_SO_4_ to each well (25 μL/well). The optical density (OD) of each well was measured at a wavelength of 450 nm using a Multiskan FC Microplate Photometer with Skanlt RE v5.0 software (Thermo Scientific, MA, USA). The endpoint serum antibody titer was determined as the highest serum dilution giving an OD higher than the mean + 3 SD of the negative control. All samples were tested in triplicate.

### Statistical analysis

Differences in endpoint antibody titers between the vaccinated and control groups were analyzed with Student’s *t*-test using GraphPad Prism v9.4.0 software for Mac (www.graphpad.com, GraphPad Software, CA, USA). All data were shown as the mean ± standard error of the mean for each experiment. p < 0.05 was considered statistically significant.

## Results

### Analysis of crude F4 fimbriae extracts

**Figure-1:**
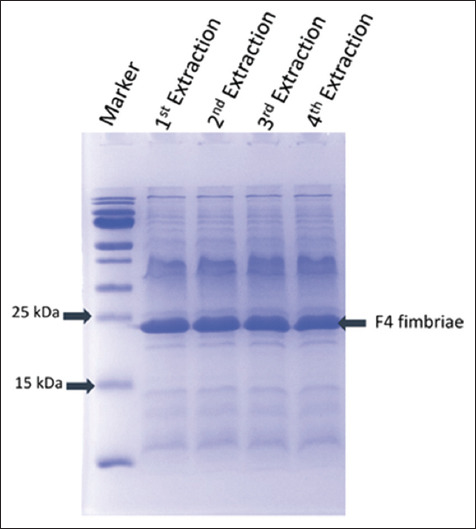
The crude F4 fimbriae extracts on a 12% gel by sodium dodecyl sulfate-polyacrylamide gel electrophoresis and Coomassie blue staining. Four lanes showed four extracts of independent extraction.

After inducing fimbriae shedding through heat shock treatment at 60°C and homogenization, the fimbriae were isolated through low-speed centrifugation. Sodium dodecyl sulfate-polyacrylamide gel electrophoresis (Figure-1) revealed the presence of minor bands of varying molecular weights and a major band of approximately 24 kDa, which corresponded to the size of FaeG, thereby confirming that they were F4 fimbriae. The total protein yield in the crude extract, as determined by the Bradford assay, was approximately 18 mg/L of culture, confirming that most of the fimbriae had been released into the suspension. In addition, LC-MS/MS confirmed that the ~24 kDa band was indeed FaeG protein. Therefore, we hypothesized that the FaeG protein prepared using our method might be of sufficient quality for use in further vaccine challenges.

### Levels of specific IgG and IgA antibodies in pig serum

**Figure-2:**
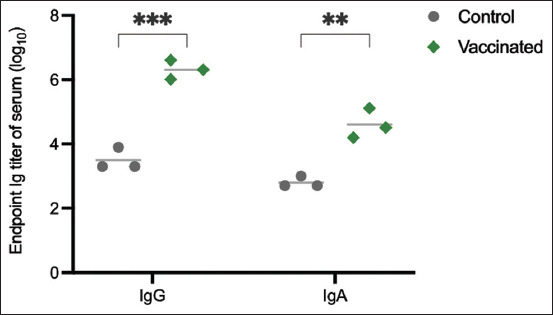
The level of serum immunoglobulin (Ig)G and IgA titer measured by enzyme-linked immunosorbent assay against F4 fimbriae antigen. Asterisks indicate a significant difference between the given groups (**means p ≤ 0.01, ***p ≤ 0.001).

**Figure-3:**
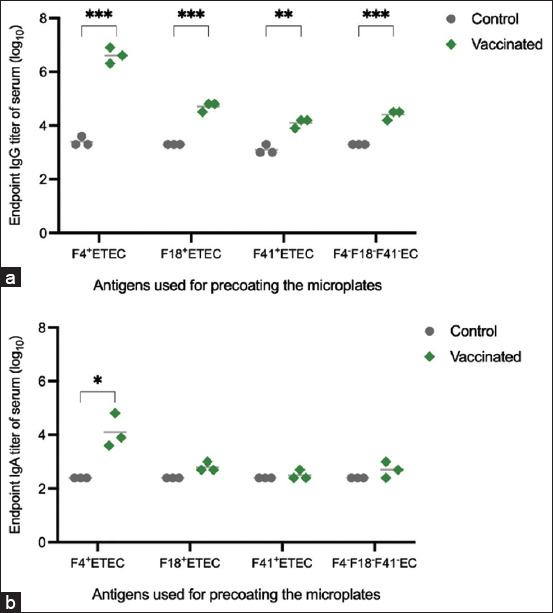
Evaluating immunoglobulin (Ig)G and IgA antibodies titer of pig serum with different *Escherichia* *coli* strains. (a) A scatter graph of endpoint IgG titers tendency against inactivated whole-cell *E. coli* strains, (b) A scatter graph of endpoint IgA titers tendency against inactivated whole-cell *E. coli* strains. Asterisks indicate a significant difference between the given groups (**means *p* ≤ 0.01, ***p ≤ 0.001).

Figure-2 shows the results of an indirect ELISA used to evaluate the anti-F4 fimbriae IgG and IgA titers in the serum samples of the vaccinated and unvaccinated groups using F4 fimbriae as the coating antigen. Piglets immunized with F4 had statistically significantly higher serum IgG and IgA titers compared with the unvaccinated group (p < 0.001 for IgG; p < 0.01 for IgA). The IgG titer of serum samples from piglets vaccinated with F4 was significantly higher than the control group against F18^+^ ETEC (p < 0.001), F4^−^F18^−^F41^−^ EC (p < 0.001), and F41^+^ ETEC (p < 0.01) antigens, indicating cross-immunity (Figure-3a). However, only the serum anti-F4^+^ ETEC IgA titer in the vaccinated group was significantly higher than in the unvaccinated control group (p < 0.01) (Figure-3b).

## Discussion

**Figure-4:**
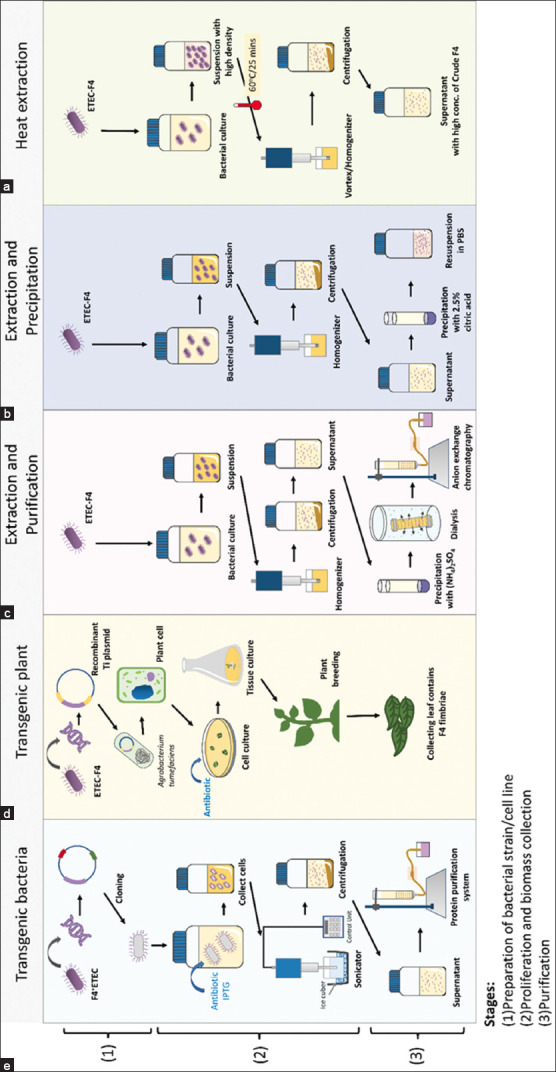
Schematic of methods of F4 fimbriae preparation for developing vaccine; (a) Transgenic bacteria, (b) Transgenic plant, (c) Extraction and purification, (d) Extraction and precipitation, and (e) Heat extraction.

In pig farming, due to economic losses, neonatal diarrhea and PWD caused by ETEC are major concerns. Among the ETEC strains, F4**^+^** ETEC is the most common and causes diarrhea associated with high morbidity and mortality in piglets [20]. Instead of antibiotics, a vaccination program may control diarrhea and decrease antibiotic resistance in pig farms. F4 fimbriae stimulate an F4-specific mucosal and systemic immune response, making them promising candidates for vaccine development [17, 21]. FaeG, a major subunit of F4 fimbriae and an adhesin, induces an immune response. Various techniques have been used to produce F4 fimbriae, such as bacterial gene transfer (Figure-4a), plant gene transfer (Figure-4b), extraction and purification (Figure-4c), and extraction and precipitation (Figure-4d). Table-1 [6, 9, 17, 21–25] presents an overview of F4 fimbriae preparation methods for vaccines. Several methods have also been used to collect fimbriae, including heat shock and homogenization methods [9, 16, 17, 21, 26]. However, the efficacy of these methods is limited when using low concentrations of bacterial suspension (3 × 10^9^ CFU/mL), resulting in crude extracts with a low concentration that require further precipitation to increase the concentration. To overcome this limitation, we modified and established a new protocol that used a bacterial suspension concentrated 10 times higher than previous methods to collect crude F4 fimbriae in high concentrations suitable for vaccine production (~300 μg/mL). We also evaluated the efficiency of the immune response stimulation of our crude fimbriae F4 extracts in nursery pigs. To the best of our knowledge, this is the first report on the results of an *E*. *coli* vaccine using crude F4 fimbriae extracts with this protocol (Figure-4e).

**Table-1:** The summarize of methods of F4 fimbriae preparation for developing vaccine.

Methods of F4 fimbriae preparation for vaccine	Transgenic bacteria	Transgenic plant	Extraction and purification	Extraction and Precipitation	Heat extraction
Fimbriae	Purified FaeG (Pure F4 fimbriae)	Leaf contain FaeG	Purified FaeG (Pure F4 fimbriae)	Suspension of precipitated F4 fimbriae extract	Crude F4 fimbriae extract
Purity	High/Very high	Low	High/Very high	Medium	Medium
Technology	High	High	Medium	Simple	Simple
Immune response	Significant	Significant	Significant	Significant	Significant
Cross-immunity	N/A	N/A	N/A	No cross-immunity (between F4 fimbriae and LT)	F18^+^ETEC, F41^+^ETEC, F4^-^F18^-^F41^-^EC
References	[6, 22, 23]	[24, 25]	[17, 21]	[9]	This study

LT=Heat-labile toxin, ETEC=Enterotoxigenic *Escherichia coli*, F18^+^ETEC=F18^-^positive ETEC, F41^+^ETEC=F41^-^positive ETEC, F4^-^F18^-^F41^-^EC=Non-fimbriae detected *E. coli*, N/A=Not analyzed

The SDS-PAGE analysis of the crude F4 fimbriae extract showed that the main component was a protein with a molecular weight of ~24 kDa, which was identified as FaeG, the major subunit of F4 fimbriae. This result is consistent with the molecular weight reported by Neidhardt (~23–23.6 kDa) [12], Pereira *et al*. (~27.5 kDa) [27], and Vandamme *et al*. (~27 kDa) [21]. Notably, the crude F4 fimbriae extract also contained other proteins with different molecular weights along with the FaeG protein. The ELISA results showed that the crude F4 fimbriae extract induced a remarkable immune response in the vaccinated group compared with the unvaccinated group. The levels of specific IgG and IgA antibodies against F4**^+^** ETEC, including isolated F4 fimbriae and whole-cell F4**^+^** ETEC, were significantly higher in the vaccinated group than in the unvaccinated group (p < 0.001 for IgG and p < 0.01 for IgA). This finding is consistent with studies by Lin *et al*. [9] and Zhang *et al*. [6].

Our study used a crude extract of F4 fimbriae from F4**^+^** ETEC as the principal component in a vaccine for pigs. The FaeG protein was present in the vaccine. The vaccinated piglets had significantly higher levels of anti-F4**^+^** ETEC antibodies than antibodies against other tested strains of *E*. *coli*. The levels of IgG and IgA antibodies in serum have been reported as related to the route of vaccine administration, with IgG levels being higher than IgA levels when vaccines were administered intramuscularly [28]. Both IgG and IgA are crucial Ig classes in the immune response system, particularly in the passive immune system, and are responsible for preventing neonatal diarrhea caused by ETEC in piglets [17]. Immunoglobulin A is crucial in the intestinal mucosal immune response against PWD caused by ETEC in piglets [9]. Immunoglobulin A antibody levels are generally high following oral or intranasal vaccine administration [21]. In our study, the IgA titer did not significantly affect the recognition of non-F4**^+^** ETEC strains (F18**^+^**, F41**^+^**, and F4^−^F18^−^F41^−^ EC). However, the level of serum IgA was significantly higher than the unvaccinated control (p < 0.01). This may be attributed to the MONTANIDE ISA 206 adjuvant used in the vaccine, which has been shown to enhance IgA immune response [29]. FaeG may also play a key role in inducing an immune response, resulting in significant differences in IgA antibodies against F4**^+^** ETEC compared with the unvaccinated control. However, our study did not directly show the mucosal IgA levels. It is well accepted that the homing of circulating IgA in pig serum occurs either when the mucosal effector sites are stimulated by a pathogen or without stimulation. To clarify, Externest *et al*. [30] showed significant relationships between serum IgA and the presence of secretory IgA when the mucosal effector sites were stimulated. Therefore, in our study, the serum IgA level may predict the release of secretory IgA (mucosal IgA level) in mucosal tissues. Overall, these findings suggest that a F4 fimbriae-based vaccine with an appropriate adjuvant can induce a significant immune response against F4^+^ ETEC, which could help to reduce the incidence of diarrhea in piglets.

Our study shows that administering a crude extract of F4 fimbriae through injection stimulates specific immune responses against F4**^+^** ETEC and also induces cross-immunity against other pathogenic *E*. *coli* strains, including F18**^+^** ETEC, F41**^+^** ETEC, and F4^−^F18^−^F41^−^ EC. Although the IgG antibody levels against other pathogenic *E*. *coli* strains were not as high as those against the F4 extract in the vaccinated group, they were significantly higher than in the unvaccinated control group (p < 0.001 for F18+ ETEC and F4^−^F18^−^F41^−^ EC; p < 0.01 for F41^+^ ETEC IgG).

**Figure-5:**
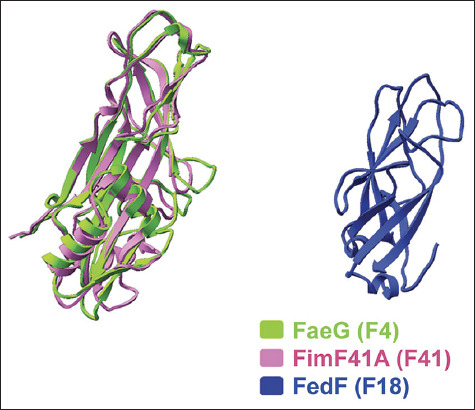
Protein structures of FaeG protein (F4), FimF41A protein (F41) and FedF protein (F18).

To the best of our knowledge, this study is the first to demonstrate that crude F4 fimbriae can stimulate immune responses against multiple *E*. *coli* strains, as demonstrated by the serum IgG titer. Crude F4 fimbriae extracts contain not only FaeG (a major subunit of F4 fimbriae) but also other colonization factors and lipopolysaccharides in addition to proteins that likely play essential roles in inducing cross-immune responses in pigs [31]. These components are either absent or exist in small amounts in vaccines comprising recombinant F4 fimbriae (FaeG) or high-purity F4 fimbriae. Although further field experiments are necessary, our findings suggest that crude F4 extract could stimulate cross-immunity. The structure of FaeG protein (F4) is highly similar to that of FimF41A (F41), while that of FedF (F18) is quite different from these two (Figure-5). However, the immune response levels of both IgG and IgA did not exhibit a clear correlation with the similarities in protein structure, suggesting that the cross-immunity detected in the serum of piglets vaccinated with our crude F4 extract may be unrelated to consensus features of fimbriae proteins and rather induced by other components of the *E*. *coli* cell that are co-extracted with the fimbriae. Crude extract-induced cross-immunization can be advantageous in inducing broader protection against ETEC in piglets, thereby reducing the risk of infection. These findings highlight the potential of crude F4 fimbriae as a candidate for the development of vaccines against diarrhea caused by F4**^+^** ETEC in pigs and were confirmed by the superior antibody titer found in piglets vaccinated with crude F4 fimbriae. However, to investigate the protective immunity of those antibodies, challenge experiments need to be performed in nursery pigs in future studies. Furthermore, another experiment should be performed in which pregnant sows are injected intramuscularly with crude F4 extract vaccine at both 10 and 12 weeks of pregnancy to investigate the correlation between IgA and IgG levels in sow serum, colostrum, and milk with those in piglet serum. Such an experiment may indicate that the serum levels of IgA and IgG home to the mucosal tissue of the udder and subsequently pass to the suckling piglet.

## Conclusion

Our study demonstrated that crude F4 fimbriae extract induces a specific immune response against F4^+^ ETEC in pigs, as evidenced by the significant increase in serum IgG and IgA levels in vaccinated piglets. Furthermore, our results suggest that crude F4 fimbriae extract can stimulate cross-immunity against other *E*. *coli* strains, including F18^+^ ETEC, F41^+^ ETEC, and F4^−^F18^−^F41^−^ EC. We propose that our simple extraction method for producing crude fimbriae extracts be exploited as an antigenic determinant for effective vaccine development to protect pigs against diarrhea caused by *E*. *coli*. This will have important implications for the pig farming industry and contribute to the development of more efficient and cost-effective vaccines.

## Authors’ Contributions

LTYN: Performed the experiments, data collection and analysis, and wrote the first manuscript. PO: Designed and supervised the study. KK: Conceptualized and supervised the study, clinical trial, edited the manuscript, and secured funding. NN: Designed and supervised the study, conceived the idea, investigated the study, analyzed data, and edited the manuscript. All authors have read, reviewed, and approved the final manuscript.

## References

[1] Hartadi E.B, Effendi M.H, Plumeriastuti H, Sofiana E.D, Wibisono F.M, Hidayatullah A.R (2020). A review of enterotoxigenic *Escherichia coli* infection in piglets:Public health importance. Syst. Rev. Pharm.

[2] Melkebeek V, Goddeeris B.M, Cox E (2013). ETEC vaccination in pigs. Vet. Immunol. Immunopathol.

[3] Kaewchomphunuch T, Charoenpichitnunt T, Thongbaiyai V, Ngamwongsatit N, Kaeoket K (2022). Cell-free culture supernatants of *Lactobacillus* spp. and *Pediococcus* spp. inhibit growth of pathogenic *Escherichia coli* isolated from pigs in Thailand. BMC Vet. Res.

[4] Keeratikunakorn K, Kaewchomphunuch T, Kaeoket K, Ngamwongsatit N (2023). Antimicrobial activity of cell free supernatants from probiotics inhibits against pathogenic bacteria isolated from fresh boar semen. Sci. Rep.

[5] Dubreuil J.D (2017). Enterotoxigenic *Escherichia coli* and probiotics in swine:What the bleep do we know?. Biosci. Microbiota Food Health.

[6] Zhang H, Xu Y, Zhang Z, You J, Yang Y, Li X (2018). Protective immunity of a multivalent vaccine candidate against piglet diarrhea caused by enterotoxigenic *Escherichia coli* (ETEC) in a pig model. Vaccine.

[7] Verdonck F, Cox E, Goddeeris B.M (2004). F4 fimbriae expressed by porcine enterotoxigenic *Escherichia coli* an example of an eccentric fimbrial system?. J. Mol. Microbiol. Biotechnol.

[8] Jeffrey J.Z (2019). Diseases of Swine.

[9] Lin J, Mateo K.S, Zhao M, Erickson A.K, Garcia N, He D, Moxley R.A, Francis D.H (2013). Protection of piglets against enteric colibacillosis by intranasal immunization with K88ac (F4ac) fimbriae and heat labile enterotoxin of *Escherichia coli*. Vet. Microbiol.

[10] García V, Gambino M, Pedersen K, Haugegaard S, Olsen J.E, Herrero-Fresno A (2020). F4-and F18-positive enterotoxigenic *Escherichia coli* isolates from diarrhea of postweaning pigs:Genomic characterization. Appl. Environ. Microbiol.

[11] Luise D, Lauridsen C, Bosi P, Trevisi P (2019). Methodology and application of *Escherichia coli* F4 and F18 encoding infection models in post-weaning pigs. J. Anim. Sci. Biotechnol.

[12] Neidhardt F.C (1996). *Escherichia coli* and *Salmonella*:Cellular and Molecular Biology.

[13] Isaacson R.E, Dean E.A, Morgan R.L, Moon H.W (1980). Immunization of suckling pigs against enterotoxigenic *Escherichia coli*-induced diarrheal disease by vaccinating dams with purified K99 or 987P pili:Antibody production in response to vaccination. Infect. Immun.

[14] Lu T, Moxley R.A, Zhang W (2019). Mapping the neutralizing epitopes of enterotoxigenic *Escherichia coli* K88 (F4) fimbrial adhesin and major subunit FaeG. Appl. Environ. Microbiol.

[15] Nguyet L.T.Y, Keeratikunakorn K, Kaeoket K, Ngamwongsatit N (2022). Antibiotic-resistant *Escherichia coli* from diarrheic piglets from pig farms in Thailand that harbor colistin-resistant *mcr* genes. Sci. Rep.

[16] Vazquez F, González E.A, Garabal J.I, Valderrama S, Blanco J, Baloda S.B (1996). Development and evaluation of an ELISA to detect *Escherichia coli* K88 (F4) fimbrial antibody levels. J. Med. Microbiol.

[17] Van den Broeck W, Cox E, Goddeeris B.M (1999). Induction of immune responses in pigs following oral administration of purified F4 fimbriae. Vaccine.

[18] Bradford M.M (1976). A rapid and sensitive method for the quantitation of microgram quantities of protein utilizing the principle of protein-dye binding. Anal. Biochem.

[19] Arshadi N, Mousavi S.L, Amani J, Nazarian S (2020). Immunogenic potency of formalin and heat-inactivated *E. coli* O157:H7 in mouse model administered by different routes. Avicenna J. Med. Biotechnol.

[20] Dubreuil J.D, Isaacson R.E, Schifferli D.M (2016). Animal enterotoxigenic *Escherichia coli*. EcoSal. Plus.

[21] Vandamme K, Melkebeek V, Cox E, Remon J.P, Vervaet C (2011). Adjuvant effect of Gantrez®AN nanoparticles during oral vaccination of piglets against F4+enterotoxigenic *Escherichia coli*. Vet. Immunol. Immunopathol.

[22] Duan Q, Ya F, Zhu G (2012). Major virulence factors of enterotoxigenic *Escherichia coli* in pigs. Ann. Microbiol.

[23] Jabif M.F, Gumina E, Hall J.W, Hernandez-Velasco X, Layton S (2021). Evaluation of a novel mucosal administered subunit vaccine on colostrum IgA and serum IgG in sows and control of enterotoxigenic *Escherichia coli* in neonatal and weanling piglets:Proof of concept. Front. Vet. Sci.

[24] Joensuu J.J, Kotiaho M, Riipi T, Snoeck V, Palva E.T, Teeri T.H, Lång H, Cox E, Goddeeris B.M, Niklander-Teeri V (2004). Fimbrial subunit protein FaeG expressed in transgenic tobacco inhibits the binding of F4ac enterotoxigenic *Escherichia coli* to porcine enterocytes. Transgenic Res.

[25] Joensuu J.J, Verdonck F, Ehrström A, Peltola M, Siljander-Rasi H, Nuutila A.M, Oksman-Caldentey K.M, Teeri T.H, Cox E, Goddeeris B.M, Niklander-Teeri V (2006). F4 (K88) fimbrial adhesin FaeG expressed in alfalfa reduces F4+enterotoxigenic *Escherichia coli* excretion in weaned piglets. Vaccine.

[26] Sarrazin E, Bertschinger H.U (1997). Role of fimbriae F18 for actively acquired immunity against porcine enterotoxigenic *Escherichia coli*. Vet. Microbiol.

[27] Pereira D.E, Vidotto M.C, Nascimento K.A, Santos A.C.R.D, Mechler M.L, Oliveira L.G.D (2016). Virulence factors of *Escherichia coli* in relation to the importance of vaccination in pigs. Cienc. Rural.

[28] Abbas A.K, Lichtman A.H, Pillai S (2015). Cellular and Molecular Immunology.

[29] Crisci E, Fraile L, Moreno N, Blanco E, Cabezón R, Costa C, Mussá T, Baratelli M, Martinez-Orellana P, Ganges L, Martínez J, Bárcena J, Montoya M (2012). Chimeric calicivirus-like particles elicit specific immune responses in pigs. Vaccine.

[30] Externest D, Meckelein B, Schmidt MA, Frey A (2000). Correlations between antibody immune responses at different mucosal effector sites are controlled by antigen type and dosage. Infect. Immun.

[31] Duan Q, Pang S, Wu W, Jiang B, Zhang W, Liu S, Wang X, Pan Z, Zhu G (2020). A multivalent vaccine candidate targeting enterotoxigenic *Escherichia coli* fimbriae for broadly protecting against porcine post-weaning diarrhea. Vet. Res.

